# Antioxidants, anti-inflammatory, and antidiabetic effects of the aqueous extracts from *Glycine* species and its bioactive compounds

**DOI:** 10.1186/s40529-016-0153-7

**Published:** 2016-11-23

**Authors:** Shyh-Shyun Huang, Shan-Yu Su, Jui-Shu Chang, Hung-Jen Lin, Wen-Tzu Wu, Jeng-Shyan Deng, Guan-Jhong Huang

**Affiliations:** 1 School of Pharmacy, College of Pharmacy, China Medical University, Taichung, Taiwan; 2 Department of Chinese Medicine, China Medical University Hospital, Taichung, Taiwan; 3 School of Chinese Medicine, Graduate Institute of Integrated Medicine College of Chinese Medicine, China Medical University, Taichung, Taiwan; 4 Department of Health and Nutrition Biotechnology, Asia University, Taichung, 413 Taiwan; 5 Department of Chinese Pharmaceutical Sciences and Chinese Medicine Resources, China Medical University, Taichung, Taiwan

**Keywords:** *Glycine* species, Antioxidant, Anti-inflammatory, α-Glucosidase, Aldose reductase

## Abstract

**Background:**

The aim of this study was to examine the possible antioxidant, anti-inflammatory, and antidiabetic effects of the aqueous extracts from three *Glycine* species. In HPLC analysis, the chromatograms of three *Glycine* species were established. Flavonoid-related compounds might be important bioactive compounds in *Glycine* species.

**Results:**

The results showed that the aqueous extract of *Glycine tabacina* (AGTa) had the strongest antioxidant activity compared with the other *Glycine* species extracts. We also found that AGTa had higher contents of total polyphenol compounds and flavonoids than the other extracts. We also have investigated the anti-inflammatory effects of the three *Glycine* species using lipopolysaccharide (LPS)-stimulated mouse macrophage (RAW264.7) ex vivo. When RAW264.7 macrophages were treated with different concentrations of three *Glycine* species together with LPS, a significant concentration-dependent inhibition of NO production was detected. The aqueous extract of *Glycine max* (AGM) had the strongest anti-inflammatory activity in comparison with the other *Glycine* species extracts. Western blotting revealed that three *Glycine* species blocked protein expression of iNOS and cyclooxygenase-2 (COX-2) in LPS-stimulated RAW264.7 macrophages, significantly. The antidiabetic activities of the three *Glycine* species were studied in vitro using α-glucosidase and aldose reductase (AR) inhibitory methods. AGTa had the highest inhibitory activities on α-glucosidase and aldose reductase, with IC_50_ of 188.1 and 126.42 μg/mL, respectively. The bioactive compounds, genistein and daidzein, had high inhibitory activities on antioxidant, anti-inflammatory, α-glucosidase and aldose reductase.

**Conclusions:**

These results suggest that *Glycine* species might be a good resource for future development of antioxidant, anti-inflammatory and antidiabetic heath foods.

## Background

Reactive oxygen species (ROS) and reactive nitrogen species have implicated in mediating various pathological processes such as cancer, aging, cardiovascular disease, neuro- degenerative diseases, and diabetic complications (Huang et al. 2014). Glycation may be important sources of radical production. Evidences reveal that many biochemical pathways associated with hyperglycaemia can increase the production of ROS (Huang et al. 2011). Glycation, the nonenzymatic reaction of reducing sugars with amino groups, leading to an acceleration of the formation of advanced glycation end products (AGEs) (Luo et al. 2016). Increased accumulation of AGEs can induce multiple cellular changes leading to macro- and micro-vascular complications. Antioxidants inhibit the glycation processes and play a theoretical strategy for preventing diabetic complications (Hsieh et al. 2010). In addition, recent studies have shown that compounds with combined antioxidant and antiglycation properties are more effective in treating diabetes mellitus (Ahmad and Ahmed 2006). α-Glucosidase play an important role in the digestion of carbohydrates in the body suppress postprandial hyperglycemia and could be useful for treating diabetic and/or obese patients (Jeon et al. 2013). Aldose reductase (AR) catalyzes the conversion of glucose to sorbitol as the first step in the polyol pathway and plays an important role in the development of some degenerative complications of diabetes (Fatmawati et al. 2014). In a variety of diabetic target tissues, AR also linked to pro-inflammatory responses and alleviated ocular inflammatory responses such as cytokines secretion. Thus, AR inhibitors have attracted attentions in therapeutic researches of diabetic complications.

Inflammation, a physiological response to infection or injury, plays a critical role in chronic diseases, including asthma, rheumatoid arthritis, atherosclerosis, and Alzheimer’s disease, and it plays a role in various human cancers (Shia et al. 2016). Among its mediators, inducible nitric oxide synthase (iNOS) and cyclooxygenase-2 (COX-2) are important enzymes that regulate inflammatory processes (Li et al. 2016). In addition, macrophages play a central role in the development of vascular inflammation and the formation of atheroma. The activity of macrophages is higher in diabetic population than that in healthy subjects (Lu et al. 2015). These findings accentuate the pathogenetic role of high glucose in macrophage activation during the process of vascular inflammation.

The *Glycine* genus, known as ‘I-Tiao-Gung’ in Chinese, is distributed in tropical areas. The traditional usages of the roots of *Glycine* species have been for the treatment of rheumatism, arthropathy, leucorrhea, menalgia, menopausal syndrome, chronic nephritis, and improvement of bone mineral density (Li et al. 2008). *Glycine tomentella* (GT) has been used in the Kinmen area of Taiwan as an anti-inflammatory agent for the treatment of rheumatic illness for a long time. Recently in Taiwan, herbal tea of GT root has been developed for commercial purposes. It has been reported that the roots of GT has several biological activities, such as antioxidant, anti-inflammatory (Chen and Pan 2007), hypolipidaemic (Ko et al. 2004), and immunomodulatory (Chuang et al. 2008). The objective of this work was to investigate the antioxidant, anti-inflammatory, and antidiabetic properties of the aqueous extracts of *Glycine max* (L.) Merr. (GM), *Glycine tomentella* Hayata (GT), and *Glycine tabacina* (Lab.) Benth (GTa) by comparing them with bioactive compounds such as genistein, and daidzein. Besides, the other objective was to find out the levels of their inhibitory activities on α-glucosidase and aldose reductase through a series of in vitro tests.

## Methods

### Materials

Glutathione (GSH), 1, 1-diphenyl-2-picrylhydrazyl radicals (DPPH), 2, 2′-azinobis-(3-ethylbenzothiazoline)-6-sulphonic acid (ABTS), genistein, genistin, daizein, daizin, and other chemicals were purchased from Sigma Chemical Co. (St. Louis, MO, USA). Folin-Ciocalteu’s phenol reagent was purchased from Merck Co. (Santa Ana, CA, USA). Anti-iNOS, anti-COX-2, and anti-β-actin antibody (Santa Cruz, USA) and a protein assay kit (Bio-Rad Laboratories Ltd., Watford, Herts, UK) were obtained as indicated. Poly-(vinylidene fluoride) membrane (Immobilon-P) was obtained from Millipore Corp. (Bedford, MA, USA). Plant materials were collected from Taichung, Nantou, and Hsinchu counties in Taiwan. They were identified and authenticated by Dr. Shyh-Shyun Huang, Department of Pharmacy, China Medical University, Taichung, Taiwan.

### Preparing aqueous extracts of plant materials

Dried herb roots (100 g for each species) were boiled with 1 L distilled water for 1 h. Filtrate and collection of the extracts were done three times. The filtrate was concentrated to a powder by freeze dryer (Christ Alpha, Germany) and stored at −20 °C.

### Chemical compositional analysis of flavonoid-related compounds and the three *Glycine* species by HPLC

HPLC was performed with a Hitachi Liquid Chromatograph (Hitachi Ltd., Tokyo, Japan). HPLC samples (10 mg/mL) were filtered through a 0.45 μm PVDF-filter and injected into the HPLC column. The injection volume was 10 μL and the separation temperature was 25 °C. The column was a Mightysil RP-18 GP (5 μm, 250 × 4.6 mm I.D.). The method involved the use of a binary gradient with mobile phases containing: (A) phosphoric acid in water (0.1%, v/v) and (B) H_2_O/Methanol: 20/80 (v/v). The solvent gradient elution program was as follows: 0–15 min, 100–75% A, 0–25% B; 15–50 min, 75–25% A, 25–75% B; and finally 50–60 min, 25–0% A, 75–100% B. The flow-rate was kept constant at 0.8 mL/min. A precolumn of μ-Bondapak^TM^ C18 (Millipore, Milford, MA, USA) was attached to protect the analytical column. Pure compounds, including daidzin, genistin, daidzein, genistein, and butyl *p*-hydroxybenzoate (internal control) were also analyzed using HPLC under the same conditions, and the retention time was used to identify the daidzin, genistin, daidzein, and genistein in the samples.

### Determining antioxidant activities by ABTS^+^ scavenging ability

The ABTS^+^ scavenging ability was determined (Chiu et al. 2013). Aqueous solution of ABTS (7 mM) was oxidized with potassium peroxodisulfate (2.45 mM) for 16 h in the dark at room temperature. The ABTS^+^ solution was diluted with 95% ethanol to an absorbance of 0.75 ± 0.05 at 734 nm (Beckman UV–Vis spectrophotometer, Model DU640B). For each sample, an aliquot (20 μL) of sample (125 μg/mL) was mixed with 180 μL ABTS^+^ solution, and then the absorbance was read at 734 nm after 1 min. Trolox was used as a reference standard.

### Determining antioxidant activity by DPPH radical scavenging ability

The effects of crude extracts and positive controls (GSH, genistein, and daidzein) on DPPH radicals were estimated (Huang et al. 2010). Aliquots of crude extracts (20 μL) at various concentrations were each mixed with 100 mM Tris–HCl buffer (80 μL, pH 7.4) and then with 100 μL of DPPH in ethanol to a final concentration of 250 μM. All the mixtures were shaken vigorously and left to stand at room temperature for 20 min in the dark. The absorbance of the reaction solutions were measured spectrophotometrically at 517 nm. The DPPH decolorizations of the samples were calculated in percentage according to the equation:  % decolorization = [1 − (ABS_sample_/ABS_control_)] × 100. IC_50_ value was the effective concentration in which DPPH 50% of radicals were scavenged and was obtained by interpolation with linear regression analysis.

### Determination of total polyphenol content

The total polyphenol content of the crude extracts were determined (Chiu et al. 2013). For each sample, 20 μL of the extract was added to 200 μL distilled water and 40 μL of Folin–Ciocalteu reagent. The mixture was allowed to stand at room temperature for 5 min, and then 40 μL of 20% sodium carbonate was added to the mixture. The resulting blue complex was measured at 680 nm. Catechin was used as a standard for the calibration curve. The polyphenol content was calibrated using the calibration curve based linear equation. The total polyphenol content was expressed as mg catechin equivalent/g dry weight. The dry weight indicated was the sample dry weight.

### Determination of total flavonoid content

The total flavonoid contents of the crude extracts were determined (Chiu et al. 2013). For each sample, an aliquot of 1.5 mL extract was added to an equal volume of 2% AlCl_3_·6H_2_O solution. The mixtures were vigorously shaken, and the absorbances at 430 nm were read after 10 min of incubation. Rutin was used as the standard for the calibration curve. The total flavonoid content was calibrated using the linear equation based on the calibration curve. The total flavonoid content was expressed as mg rutin equivalent/g dry weight. The dry weight indicated was the sample dry weight.

### Cell culture

A murine macrophage cell line RAW264.7 (BCRC No. 60001) was purchased from the Bioresources Collection and Research Center (BCRC) of the Food Industry Research and Development Institute (Hsinchu, Taiwan). Cells were cultured in plastic dishes containing Dulbecco’s Modified Eagle Medium (DMEM, Sigma, St. Louis, MO, USA) supplemented with 10% fetal bovine serum (FBS, Sigma, USA) in a CO_2_ incubator (5% CO_2_ in air) at 37 °C and subcultured every 3 days at a dilution of 1:5 using 0.05% trypsin–0.02% EDTA in Ca^2+^-, Mg^2+^- free phosphate-buffered saline (DPBS).

### Cell viability

Cells (2 × 10^5^) were cultured in 96-well plate containing DMEM supplemented with 10% FBS for 1 day to become nearly confluent. Then cells were cultured with samples in the presence of 100 ng/mL LPS for 24 or 1 h. After that, the cells were cleaned twice with DPBS and incubated with 100 μL of 0.5 mg/mL MTT for 2 h at 37 °C testing for cell viability. The medium was then discarded and 100 μL dimethyl sulfoxide (DMSO) was added. After 30-min incubation, absorbance at 570 nm was read by using a microplate reader (*Molecular Devices*, *Orleans Drive*, *Sunnyvale*, *CA, USA*).

### Measurement of nitric oxide/nitrite

NO production was indirectly assessed by measuring the nitrite levels in BALF determined by a colorimetric method based on the Griess reaction (Shie et al. 2015). 100 μL supernatant was applied to a microtiter plate well, followed by 100 μL of Griess reagent (1% sulfanilamide and 0.1% *N*-1-naphthylethylenediamine dihydrochloride in 2.5% polyphosphoric acid). After 10 min of color development at room temperature, the absorbance was measured at 540 nm with a Micro-Reader (*Molecular Devices*, *Orleans Drive*, *Sunnyvale*, *CA, USA*). By using sodium nitrite to generate a standard curve, the concentration of nitrite was measured by absorbance at 540 nm.

### Protein lysate preparation and Western Blot analysis

The stimulated murine macrophage cell line RAW264.7 cells were washed with PBS and lysed in an ice-cold lysis buffer [10% glycerol, 1% Triton X-100, 1 mM Na_3_VO_4_, 1 mM EGTA, 10 mM NaF, 1 mM Na_4_P_2_O_7_, 20 mM Tris buffer (pH 7.9), 100 mM β-glycerophosphate, 137 mM NaCl, 5 mM EDTA, and one protease inhibitor cocktail tablet (Roche, Indianapolis, IN, USA)] on ice for 1 h, followed by centrifugation at 12,000×*g* for 30 min at 4 °C. Soft tissues were removed from individual mice paws and homogenized in a solution containing 10 mM CHAPS, 1 mM phenylmethylsulphonyl fluoride (PMSF), 5 μg/mL, aprotinin, 1 μM pepstatin and 10 μM leupeptin. The homogenates were centrifuged at 12,000×*g* for 20 min, and 30 μg of protein from the supernatants was then separated on 10% sodium dodecylsulphate–polyacrylamide gel (SDS-PAGE) and transferred to polyvinylidene difluoride membranes. After transfer, the membrane was blocked for 2 h at room temperature with 5% skim milk in Tris-buffered saline-Tween (TBST; 20 mM Tris, 500 mM NaCl, pH 7.5, 0.1% Tween 20). Later, the membranes were incubated with antibody in 5% skim milk in TBST for 2 h at room temperature. The membranes were washed three times with TBST at room temperature and then incubated with a 1:2000 dilution of anti-mouse IgG secondary antibody conjugated to horseradish peroxidase (Sigma, St Louis, MO, USA) in 2.5% skim milk in TBST for 1 h at room temperature. The membranes were washed three times and the immunoreactive proteins were detected by enhanced chemiluminescence (ECL) by using hyperfilm and ECL reagent (Amersham International plc., Buckinghamshire, UK). The results of Western Blot analysis were quantified by measuring the relative intensity compared to the control by using Kodak Molecular Imaging Software (Version 4.0.5, Eastman *Kodak* Company, Rochester, NY, USA) and represented in the relative intensities.

### Inhibition assay for α-glucosidase activity

The α-glucosidase inhibitory effects of the aqueous extracts from three *Glycine* species were assayed with minor modifications (Cao et al. 2009). Briefly, the enzyme reaction was performed using *p*-Nitrophenyl-alpha-d-glucopyranoside (PNP-glycoside) as a substrate in 0.1 M piperazine- N,N′-bis (2-ethanesulfonic acid) (PIPES) buffer, pH 6.8. The PNP-glycoside (2.0 mM) was premixed with samples at various concentrations. Each mixture was added to α-glucosidase solution (0.01 units, from bakers yeast) to make 0.5 mL of final volume. The reaction was terminated by adding 1 ml of 0.64% *N*-(1-naphthyl) ethylenediamine solution (pH 10.7). Enzymatic activity was quantified by measuring the *p*-nitrophenol released from PNP-glycoside at 400 nm wave length. All reactions were carried out at 37 °C for 30 min with three replications.

### Measure aldose reductase activity in vitro

Crude aldose reductase (AR) was prepared as in the following steps: lenses were removed from Sprague–Dawley (SD) rats weighing 250–280 g, and were kept frozen until use. A homogenate of rat lens was prepared in accordance with the method described (Huang et al. 2011). A partially purified enzyme, with a specific activity of 6.5 U/mg, was routinely used in the evaluations of enzyme inhibition. The partially purified material was separated into 1.0 mL aliquots, and stored at −40 °C. The AR activity was spectrophotometrically assayed by measuring the decrease in NADPH absorption at 340 nm over a 4 min period, using DL-glyceraldehyde as a substrate. Each 1.0 mL cuvette contained equal units of enzyme, 0.10 M sodium phosphate buffer (pH 6.2) and 0.3 mM NADPH, both with and without 10 mM of the substrate and an inhibitor. Each 1.0 mL of cuvette containing equal units of enzyme, 0.1 M sodium phosphate buffer (pH 6.2), 0.3 mM NADPH with or without 10 mM substrate and inhibitor was prepared. One set of mixtures prepared with an equivalent volume of sodium phosphate buffer instead of tested samples was used as control. The concentration of the extracts required to inhibit 50% of AR activity under the assay conditions was defined as the IC_50_ value.

### Statistical analyses

Experimental results were presented as the mean ± standard deviation (SD) of three parallel measurements. The statistical analyses were performed by one-way ANOVA, followed by Dunnett’s *t* test. A difference was considered to be statistically significant when *p* < 0.05, *p* < 0.01 or *p* < 0.001.

## Results and discussion

### Compositional analyses of flavonoid-related compounds and the three *Glycine* species by HPLC

**Fig. 1:**
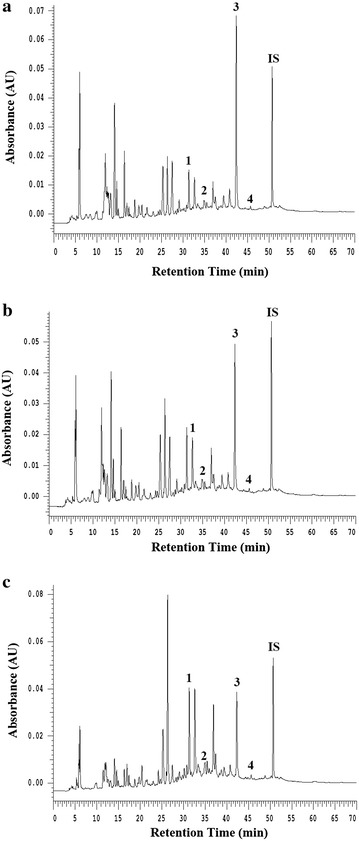
HPLC chromatogram of the aqueous of *Glycine max* (AGM) (**a**), the aqueous of *Glycine tomentella* (AGT) (**b**), and the aqueous of *Glycine tabacina* (AGTa) (**c**). The peaks indicate the following *1* daidzin; *2* genistin; *3* daidzein; *4* genistein. IS butyl p-hydroxybenzoate

To verify whether flavonoid-related compounds were presented in the three *Glycine* species, marker compounds (genistein, daidzein, genistein and daidzin*)* were separated through HPLC column separately under the same conditions. Figure 1 shows the HPLC analytical plot for the three *Glycine* species, with the four flavonoid-related components identified as daidzin (retention time, 31.8 min), genistin (35.1 min), daidzein (42.8 min) and genistein (45.9 min). Butyl *p*-hydroxybenzoate is an internal standard (IS). According to the plot of the peak-area ratio (y) vs. concentration (x, g/mL), the regression equations of the four constituents and their correlation coefficients (r) were as follows: daidzin, y = 0.0509x + 0.2852 (R^2^ = 0.9981); genistin, y = 0.0619x + 0.3388 (R^2^ = 0.9954); daidzein, y = 0.0276x + 0.0240 (R^2^ = 0.9988); genistein, y = 0.0424x + 0.0658 (R^2^ = 0.9982). The relative amounts of the four phenolic compounds found in AGM, AGT, and AGTa were in the order daidzein (6.42, 5.40 and 4.79) > daidzin (0.36, 0.80 and 1.89) > genistin (0.07, 0.16 and 0.31) > genistein (0.01, 0.02, and 0.05). The HPLC fingerprint indicated that the three *Glycine* species contained these four marker compounds.

### Antioxidant activity estimated by ABTS

ABTS assay is often used in evaluating the total antioxidant power of single compound and complex mixtures of various plants. In this assay, ABTS radical monocations were generated directly from the stable form of potassium peroxydisulfate. Radicals were generated before the antioxidants were added to prevent interference of compounds, which could have affected radical formation. This modification made the assay less susceptible to artifacts and prevented overestimation of antioxidant power (Huang et al. 2013). Antioxidant samples were added to the reaction medium when the absorbance became stable, and then the antioxidant activity was measured in terms of decolorization.

Results of the ABTS assay was expressed in TEAC value. A higher TEAC value meant that the sample had a stronger antioxidant activity. TEAC values of the three *Glycine* species were determined from the calibration curve, as shown in Table 1. Antioxidant activities of the aqueous extracts of the three *Glycine* species were in the following decreasing order: AGTa (8.47 ± 0.08 μmol TE/g) > AGT (7.76 ± 0.04 μmol TE/g) > AGM (7.67 ± 0.04 μmol TE/g). The antioxidant potency of genistein and daidzein (positive control) was 32.57 ± 0.17 and 30.52 ± 0.33 μmol TE/g, respectively.

**Table 1:** Contents of total polyphenols, flavonoids, and radical scavenging activity of the aqueous extracts from three *Glycine* species determined by TEAC, and DPPH assay

Species and positive controls	Total phenols^a^ (μg CE/mg)	Total flavonoids^b^ (μg RE/mg)	TEAC (μmol TE/g)	DPPH IC_50_ value (µg/mL)
AGM	21.58 ± 0.04	3.86 ± 0.13	7.67 ± 0.04	378.18
AGT	76.35 ± 0.09	7.43 ± 0.14	7.76 ± 0.04	249.88
AGTa	94.92 ± 0.12	7.62 ± 0.12	8.47 ± 0.08	212.41
Genistein	(−)	(−)	32.57 ± 0.17	106.22
Daidzein	(−)	(−)	30.52 ± 0.33	90.68

All values expressed as mean ± SD of triplicate tests. (*n* = 3). Means which did not share a common letter were significantly different (*p* < 0.05) when analyzed by ANOVA and Ducan’s multiple-range tests

^a^Data expressed in μg catechin equivalent/mg dry weight (μg CE/mg)

^b^Data expressed in μg rutin equivalent/mg dry weight (μg rutin/mg)

### Scavenging activity against 1,1-diphenyl-2-picrylhydrazyl radicals

The relatively stable organic DPPH radicals are widely used in model systems to investigate the scavenging activities of several natural compounds, such as phenolics and anthocyanins, or crude mixtures (Liao et al. 2013). A DPPH radical is scavenged by antioxidants through the donation of a proton to form the reduced DPPH. The color changes from purple to yellow after reduction, which could be quantified by its decrease of absorbance at wavelength 517 nm. Radical scavenging activity increases when the percentage of free radical inhibition increases. Table 1 shows the IC_50_ values for the radical-scavenging activities of the three *Glycine* species, genistein, and daidzein using the DPPH colorimetric method. It was found that AGTa had the lowest IC_50_ value (212.41 μg/mL), followed by AGT (249.88 μg/mL), and AGM (378.18 μg/mL). As demonstrated by the above results, the most active sample was AGTa, however, its antioxidant capacity was still stronger than genistein positive controls (106.22 μg/mL), but weaker than daidzein positive controls (90.68 μg/mL) in DPPH assay.

### Total polyphenol and flavonoid content

The total polyphenol, flavonoid, and flavonol contents of the three *Glycine* species were shown in Table 1. The total polyphenol content was expressed as μg of catechin equivalent per minigram of dry weight. The total polyphenol contents of the extracts of the three *Glycine* species ranged from 21.58 to 94.92 μg CE/mg, and decreased in the following order: AGTa>AGT>AGM. AGTa had the highest polyphenolic content.

The total flavonoid contents were expressed as μg of rutin equivalent per milligram of dry weight. The total flavonoid contents of the extracts of the three *Glycine* species ranged from 3.86 to 7.62 μg RE/mg, and decreased in the following order: AGTa>AGT>AGM. AGT had the highest flavonoid content.

Flavonoid is polyphenolic compounds. Polyphenolic compounds have important roles in stabilizing lipid oxidation and are associated with antioxidant activities. The phenolic compounds may contribute directly to antioxidative actions. It is suggested that 1.0 g of polyphenolic compounds from a daily diet rich in fruits and vegetables has inhibitory effects on mutagenesis and carcinogenesis in humans (Rakshamani et al. 2007). The antioxidants had different functional properties, such as ROS scavenging, e.g. quercetin, rutin, and catechin free radical generation inhibitions and chain-breaking activity, for example *p*-coumaric acids and metal chelation (Van Acker et al. 1998). These antioxidative compounds are usually phenolic compounds that are effective in donating protons, such as tocopherols, flavonoids, and other organic acids. However, the active contents responsible for the antioxidative activities of the three *Glycine* species are still unclear. Therefore, further work must be performed to isolate and identify these components.

### Cell viability and effect of the *glycine* species on LPS-induced NO production in macrophages

**Fig. 2:**
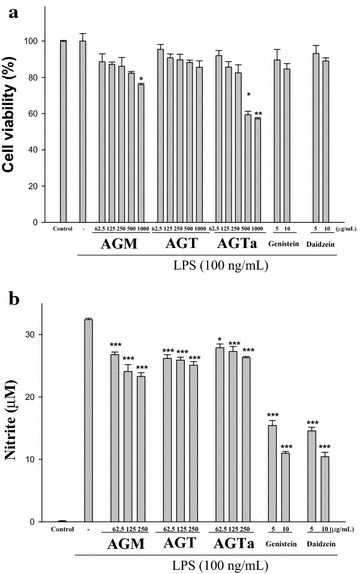
Effects of three *Glycine* species, genistein, and daidzein on lipopolysaccharide (LPS)-induced cell viability (**a)** and NO production (**b**) of RAW 264.7 macrophages. Cells were incubated for 24 h with 100 ng/mL of LPS in the absence or presence of three *Glycine* species (0, 62.5, 125, 250, 500 and 1000 μg/mL), genistein, or daidzein (5 and 10 μg/mL). Samples were added 1 h before incubation with LPS. Cell viability assay was performed using MTT assay. Nitrite concentration in the medium was determined using Griess reagent. The data were presented as mean ± SD for three different experiments performed in triplicate. ^###^Compared with sample of control group. *p < 0.05 and **p < 0.01 were compared with LPS-alone group

The effect of the three *glycine* species on RAW264.7 cell viability was determined by a MTT assay. Cells cultured with three glycine species at the concentrations (0, 62.5, 125 and 250 μg/mL) used in the presence of 100 ng/mL LPS for 24 h did not change cell viability (Fig. 2a). When RAW264.7 macrophages were treated with different concentrations of three glycine species (0, 62.5, 125 and 250 μg/mL) together with LPS (100 ng/mL) for 24 h, a significant concentration-dependent inhibition of nitrite production was detected. There was a significant decrease in the nitrite production of group treated with 250 μg/mL AGM, AGT, and AGTa when compared with the LPS-alone group (*p* < 0.001) (Fig. 2b). These data implied that AGM had the highest anti-inflammatory activity.

### Cell viability and effect of genistein and daidzein on LPS-induced NO production in macrophages

The effect of genistein and daidzein on RAW264.7 cell viability was determined by a MTT assay. Cells cultured with three glycine species at the concentrations (0, 5 and 10 μg/mL) used in the presence of 100 ng/mL LPS for 24 h did not change cell viability (Fig. 2a). When RAW264.7 macrophages were treated with different concentrations of genistein and daidzein (0, 5 and 10 μg/mL) together with LPS (100 ng/mL) for 24 h, a significant concentration-dependent inhibition of nitrite production was detected. There was a significant decrease in the nitrite production of group treated with 10 μg/mL genistein and daidzein when compared with the LPS-alone group (*p* < 0.001) (Fig. 2b).

### Inhibition of LPS-induced iNOS and COX-2 protein by three glycine species

**Fig. 3:**
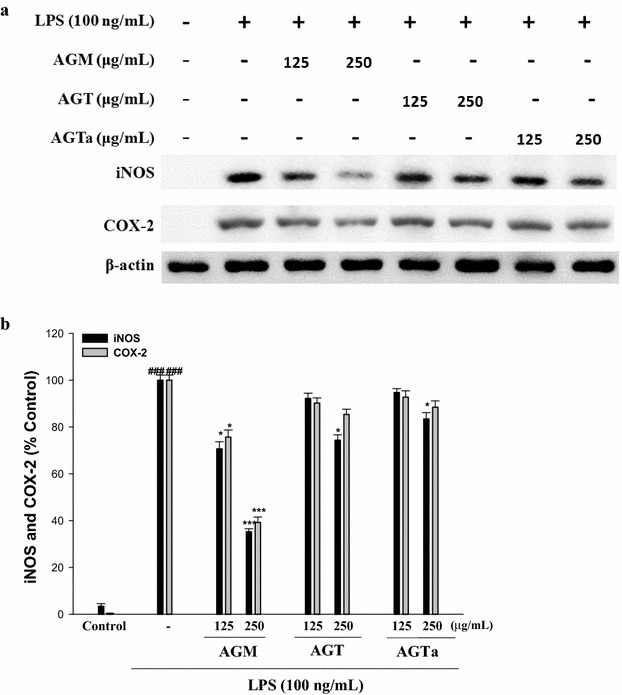
Inhibition of iNOS and COX-2 protein expression by three *Glycine* species in LPS-stimulated RAW264.7 cells. Cells were incubated for 24 h with 100 μg/mL of LPS in the absence or the presence of three *Glycine* species (0, 125 and 250 μg/mL). Samples were added 1 h before incubation with LPS. Lysed cells were then prepared and subjected to western blotting using an antibody specific for iNOS and COX-2 (**a**). The values under each lane indicate the relative band intensities normalized to β-actin (**b**). β-actin was used as a quantity control. The data were presented as mean ± SD for three different experiments performed in triplicate

In order to investigate whether the inhibition of NO production was due to a decreased iNOS and COX-2 protein level, the effect of three glycine species on iNOS and COX-2 protein expression was studied by immunoblot. The results showed that incubation with three glycine species (0, 125 and 250 μg/mL) in the presence of LPS (100 ng/mL) for 24 h inhibited iNOS and COX-2 proteins expression in mouse macrophage RAW264.7 cells in a dose-dependent manner (Fig. 3a, b). The detection of β-actin was also performed in the same blot as an internal control. The intensity of protein bands were showed an average of 64.8 and 60.8% down-regulation of iNOS and COX-2 proteins, respectively, after treatment with AGM at 250 μg/mL compared with the LPS-alone (Fig. 3b). However, AGM treatment resulted in the decrease of iNOS and COX-2 induction by LPS. The inhibition effect of iNOS and COX-2 by AGM was better than others *glycine* species (AGT and AGTa) in LPS-stimulated murine macrophages (Fig. 3).

### Effects of genistein and daidzein on LPS-Induced COX-2 and iNOS protein expressions in RAW 264.7 Cells

**Fig. 4:**
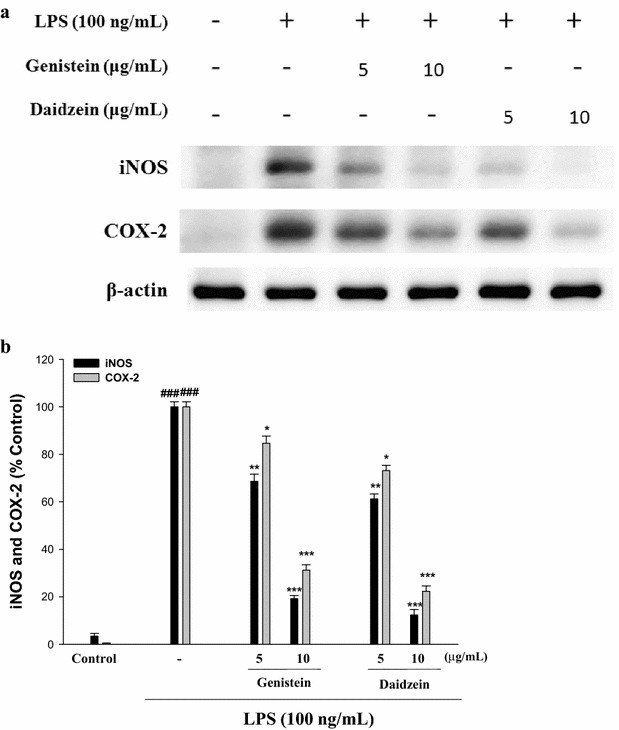
Inhibition of iNOS and COX-2 protein expression by genistein and daidzein in LPS-stimulated RAW264.7 cells. Cells were incubated for 24 h with 100 μg/mL of LPS in the absence or the presence of genistein or daidzein (5 and 10 μg/mL). Samples were added 1 h before incubation with LPS. Lysed cells were then prepared and subjected to Western Blotting using an antibody specific for iNOS and COX-2 (**a**). The values under each lane indicate the relative band intensities normalized to β-actin (**b**). β-actin was used as a quantity control. The data were presented as mean ± SD for three different experiments performed in triplicate

As shown in Fig. 4a and b, in contrast, murine macrophage cells treated with LPS alone showed dramatic inductions of COX-2 and iNOS. Cells treated with genistein and daidzein inhibited LPS-induced iNOS and COX-2 protein expressions in the LPS-induced RAW 264.7 cells. The data indicated that the inhibitory actions on iNOS and COX-2 protein expressions by genistein and daidzein may be partially responsible for the inhibition of NO formation. The intensity of protein bands were showed an average of 87.6 and 80.8% down-regulation of iNOS and COX-2 proteins, respectively, after treatment with daidzein at 10 μg/mL compared with the LPS-alone. The intensity of protein bands were showed an average of 77.6 and 68.8% down-regulation of iNOS and COX-2 proteins, respectively, after treatment with genistein at 10 μg/mL compared with the LPS-alone (Fig. 3b). Thus, daidzein decrease of iNOS and COX-2 induction better than genistein in LPS-stimulated murine macrophages (Fig. 4).

### Inhibitiory assay for α-glucosidase activity

The α-glucosidase inhibitory activity of the aqueous extracts from three *Glycine* species is shown in Table 2. The effectiveness of enzymatic inhibition of the aqueous extracts of the three *Glycine* species was determined by calculating IC_50_. The lower the value showed the higher the quality of enzymatic inhibition. The IC_50_ of the three *Glycine* species in inhibiting α-glucosidase ranged from 188.1 to 405.83 μg/mL, and its effectiveness was ranged as in the following increasing order: AGTa>AGT>AGM. AGTa had the highest α-glucosidase inhibitory activity (IC_50_ = 188.1 μg/mL). The bioactive compounds against α-glucosidase inhibitory activity were genistein (IC_50_ = 20.91 μg/mL), daidzein (IC_50_ = 13.69 μg/mL) and acarbose (IC_50_ = 517.98 μg/mL).

**Table 2:** Inhibitory effect of the aqueous extracts from three *Glycine* species on the α-glucosidase and aldose reductase inhibition

Species and positive controls	α-Glucosidase inhibitor IC_50_ value (µg/mL)	Aldose reductase inhibitor IC_50_ value (µg/mL)
AGM	405.83	244.81
AGT	211.17	148.98
AGTa	188.1	126.42
Acarbose	517.98	N.D.^a^
Genistein	20.91	42.52
Daidzein	13.69	31.95

Values represented mean ± SD of three parallel measurements

^a^ *N.D.* not detected

The IC_50_ of positive control for α-glucosidase inhibitor (acarbose) is found much higher in the present assay which is similar to many previous literatures (Shinde et al. 2008). When compared to acarbose as the control, only mammalian enzyme was inhibited. This was expected since acarbose has been shown to be a potent inhibitor of mammalian sucrase and maltase and inactive against yeast and bacterial forms (Kim et al. 2004).

Polyphenolic compounds in plants have been thought to inhibit the activities of digestive enzymes for a long time because of their ability to bind with proteins. Genistein and daidzein belongs to the isoflavonoid family. Most previous studies have focused on the pharmacological activities of genistein as a tyrosine kinase inhibitor, and its chemoprotectant activities against cancers and cardiovascular disease. In addition, genistein and daidzein could be a potent α-glucosidase and α-amylase inhibitor (Kim et al. 2000).

### Measurement of aldose reductase activity in vitro

AR, the principal enzyme of the polyol pathway, has been shown to play an important role in the complications associated with diabetes. The AR inhibitory activity of the aqueous extracts from three *Glycine* species is shown in Table 2. The IC_50_ of the extracts of the three *Glycine* species AR inhibitory activities ranged from 126.42 to 244.81 μg/mL, and increased as in the following order: AGTa>AGT>AGM. AGTa had the highest AR inhibitory activity (IC_50_ = 126.42 μg/mL). The bioactive compounds in the AR inhibitory activity assay were genistein (IC_50_ = 42.52 μg/mL) and daidzein (IC_50_ = 31.95 μg/mL).

Plant-derived extracts and phytochemicals are potential alternatives to synthetic inhibitors against AR and α-glucosidase. Currently, AR inhibitor and α-glucosidase inhibitor compounds isolated from plants are classified as diterpene-, triterpene-, and flavonoid-related compounds (Lee 2006). In this study, the active components isolated from AGTa against aldose reductase and α-glucosidase was identified as genistein and daidzein, even though the inhibitory responses varied with concentrations.

Many natural compounds have been tested for AR inhibitory activities. Medicinal plants are particularly likely to be non-toxic and may be useful for the prevention and treatment of diabetes-related complications. In addition to its antioxidant properties, genistein has an inhibitory effect on the formation of advanced glycation end products (Jang et al. 2006). Further evidence about that genistein can inhibit diabetic related problems stems from studies with type 2 diabetic animals; genistein has been shown to decrease blood glucose and glycated hemoglobin levels (HbA1C) and increase the glucagon/insulin ratio (Lee 2006). And genistein and daidzein also prevent diabetes onset by elevating insulin level and altering hepatic gluconeogenic and lipogenic enzyme activities in non-obese diabetic (NOD) mice (Choi et al. 2008).

In conclusion, the results from in vitro experiments, including HPLC assay (Fig. 1), ABTS radical monocation scavenging, DPPH radical scavenging, total polyphenol content, and total flavonoid content (Table 1), anti-inflammatory activity (Fig. 2), α-glucosidase inhibition, and AR inhibition (Table 2) demonstrated that the phytochemicals in the aqueous extracts of the three *Glycine* species might have significant antioxidant, anti-inflammatory, and anti-diabetic activities, directly related to the total amount of polyphenols and flavonoid. Hence, the three *Glycine* species could be used as easy accessible sources of natural antioxidants in pharmaceutical and medical industries. For this reason, further work could be performed to isolate and identify the antioxidant or anti-diabetic components of the GTa.

## References

[CR1] Ahmad MS, Ahmed N (2006). Antiglycation properties of aged garlic extract: possible role in prevention of diabetic complications. J Nutr.

[CR2] Cao S, Zheng Y, Yang Z, Wang K, Rui H (2009). Effect of methyl jasmonate on quality and antioxidant activity of postharvest loquat fruit. J Sci Food Agric.

[CR3] Chen TY, Pan BS (2007). Ex vivo inhibitory effect on tilapia LDL oxidation and hypolipidemia properties of *Glycine tomentella* root extract. Comp Biochem Physiol Part A Mol Integr Physiol.

[CR4] Chiu CS, Deng JS, Chang HY, Chen YC, Lee MM, Hou WC, Lee CY, Huang SS, Huang GJ (2013). Antioxidant and anti-inflammatory properties of Taiwanese Yam (Dioscorea japonica Thunb. var. pseudojaponica (Hayata) Yamam.) and its reference compounds. Food Chem.

[CR5] Choi MS, Jung UJ, Yeo J, Kim MJ, Lee MK (2008). Genistein and daidzein prevent diabetes onset by elevating insulin level and altering hepatic gluconeogenic and lipogenic enzyme activities in non-obese non-obese diabetic (NOD) mice. Diabetes/Metab Res Rev.

[CR6] Chuang WL, Haugland Ø, Pan BS, Evensen Ø (2008). Isoflavone-rich extracts from wooly glycine *Glycine tomentella* inhibits LPS-induced TNF-α expression in a macrophage cell line of Atlantic salmon (*Salmo salar* L.). Mol Immunol.

[CR7] Fatmawati S, Ersam T, Yu H, Zhang C, Jin F, Shimizu K (2014). 20(S)-Ginsenoside Rh2 as aldose reductase inhibitor from Panax ginseng. Bioorg Med Chem Lett.

[CR8] Hsieh PC, Huang GJ, Ho YL, Lin YH, Huang SS, Chiang YC, Tseng MC, Chang YS (2010). Activities of antioxidants, α-Glucosidase inhibitors and aldose reductase inhibitors of the aqueous extracts of four *Flemingia* species in Taiwan. Bot Stud.

[CR9] Huang GJ, Chiu CS, Wu CH, Huang SS, Hou WC, Amagaya S, Sheu MJ, Liao JC, Lin YH (2010). Redox status of Bowman–Birk Inhibitor from soybean Influence its in vitro antioxidant activities. Bot Stud.

[CR10] Huang GJ, Hsieh WT, Chang HY, Huang SS, Lin YC, Kuo YH (2011). Inhibitory constituents of α-glucosidase and aldose reductase in the fruiting body of *Phellinus merrillii*. J Agric Food Chem.

[CR11] Huang SS, Chiu CS, Lin TH, Lee MM, Lee CY, Chang SJ, Hou WC, Huang GJ, Deng JS (2013). Antioxidant and anti-inflammatory activities of aqueous extract of *Centipeda minima*. J Ethnopharmacol.

[CR12] Huang SS, Deng JS, Chen HJ, Lin YH, Huang GJ (2014). Antioxidant activities of two metallothionein-like proteins from sweet potato (*Ipomoea batatas* [L.] Lam. ‘Tainong 57’) storage roots and their synthesized peptides. Bot Stud.

[CR13] Jang DS, Kim JM, Lee YM, Kim YS, Kim H, Kim JS (2006). Puerariafuran, a new inhibitor of advanced glycation end products (AGEs) isolated from the roots of *Pueraria lobata*. Chem Pharm Bull.

[CR14] Jeon SY, Oh S, Kim E, Imm JY (2013). α-Glucosidase inhibiton and antiglycation activity of laccase-catalyzed catechin polymers. J Agric Food Chem.

[CR15] Kim JS, Kwon CS, Son KH (2000). Inhibiton of alpha-glucosidase and amylase by Luteolin, a flavonoid. Biosci Biotechnol Biochem.

[CR16] Kim YM, Wang MH, Rhee HI (2004). A novel α-glucosidase inhibitor from pine bark. Carbohydr Res.

[CR17] Ko YJ, Wu YW, Lin WC (2004). Hypolipidemic effect of *Glycine tomentella* root extract in Hamsters. Am J Chin Med.

[CR18] Lee JS (2006). Effects of soy protein and genistein on blood glucose, antioxidant enzyme activities, and lipid profile in streptozotocin-induced diabetic rats. Life Sci.

[CR19] Li H, Yang M, Miao J, Ma X (2008). Prenylated isoflavones from *Flemingia philippinensis*. Magnetic Reson Chem: MRC.

[CR20] Li KC, Ho YL, Chen CY, Hsieh WT, Chang YS, Huang GJ (2016). lobeline improve acute lung injury via Nuclear Factor-kB-signaling pathway and oxidative stress. Respir Physiol Neurobiol.

[CR21] Liao JC, Deng JS, Chiu CS, Huang SS, Hou WC, Lin WC, Huang GJ (2013). Chemical compositions, anti-inflammatory, antiproliferative and radical-scavenging activities of the methanol extracts of *Actinidia callosa* var. ephippioides. Am J Chin Med.

[CR22] Lu Z, Zhang X, Li Y, Lopes-Virella MF, Huang Y (2015). TLR4 antagonist attenuates atherogenesis in LDL receptor-deficient mice with diet-induced type 2 diabetes. Immunobiol..

[CR23] Luo X, Wu J, Jing S, Yan LJ (2016). Hyperglycemic stress and carbon stress in diabetic glucotoxicity. Aging Dis..

[CR24] Rakshamani T, Mohan H, Kamat JP (2007). Modulation of oxidative damage by natural products. Food Chem.

[CR25] Shia PH, Wang SY, Lay HL, Huang GJ (2016). 4, 7-dimethoxy-5-methyl-1,3-benzodioxole from *Antrodia camphorata* inhibits LPS-induced inflammation via suppression of NF- < kappa > B and induction HO-1 in RAW264.7 cells. Int. Immunopharmol..

[CR26] Shie PH, Huang SS, Deng JS, Huang GJ (2015). *Spiranthes sinensis* (Pers.) Ames. suppress production of pro-inflammatory mediators through the down-regulation of NF-κB signaling pathway and up-regulation of HO-1/Nrf2 antioxidant protein. Am J Chin Med.

[CR27] Shinde J, Taldone T, Barletta M, Kunaparaju N, Hu B, Kumar S, Placido J, Zito SW (2008). α-Glucosidase inhibitory activity of *Syzygium cumini* (Linn.) skeels seed kernel in vitro and in Goto-Kakizaki (GK) rats. Carbohydr Res.

[CR28] Van Acker SA, Van Balen GP, Van den Berg DJ, Bast A, Van der Vijgh WJ (1998). Influence of iron chelation on the antioxidant activity of flavonoids. Biochem Pharmacol.

